# Sleep Disturbances After General Anesthesia: Current Perspectives

**DOI:** 10.3389/fneur.2020.00629

**Published:** 2020-07-08

**Authors:** Man Luo, Bijia Song, Junchao Zhu

**Affiliations:** ^1^Department of Anesthesiology, Shengjing Hospital of China Medical University, Shenyang, China; ^2^Department of Anesthesiology, Friendship Hospital of Capital Medical University, Beijing, China

**Keywords:** general anesthesia, sleep, sleep disturbance, adverse effects, therapy

## Abstract

The purpose of this article is to review (1) sleep mechanism under general anesthesia, harmful effects of postoperative sleep disturbances; (2) risk factors associated with postoperative sleep disturbances; (3) measures to prevent and improve postoperative sleep disturbances. General anesthesia changes the postoperative sleep structure especially in elderly patients after major surgery and results in a high incidence rate of sleep disturbances. Sleep disturbances produce harmful effects on postoperative patients and lead to a higher risk of delirium, more cardiovascular events, and poorer recovery. Some researchers do propose non-pharmacological treatments such as attention to environmental and psychological factors, application of electroacupuncture (EA) technology and pharmacological treatments are helpful, but larger high-quality clinical trials with longer following-up are needed to further investigate the efficacy and safety.

## Introduction

General anesthesia is a medically induced state of low reactivity consciousness (1). Given the agent-specific clinical differences, general anesthesia is not considered a unitary state but rather an aggregate of the following discrete clinical endpoints: hypnosis (unawareness of one's environment), analgesia (lack of pain sensitivity), amnesia (lack of memory), and immobility after surgical stimulation (2). Current studies have revealed that sleep disturbances frequently occur in patients after surgery under general anesthesia. Postoperative sleep disturbances are not only one of the manifestations of postoperative brain dysfunction (3), but also can induce postoperative fatigue, metabolic disorders, hypertension, cerebrovascular, and cardiovascular disease. Besides, it is considered an important risk factor for delirium development (4). Here we reviewed pieces of evidence regarding sleep mechanism under general anesthesia, sleep rhythm disorders, risk factors, and treatments of postoperative sleep disturbances.

## Sleep Mechanism and Circadian Rhythm Under General Anesthesia

The spontaneous sleep-wake cycle is repeated every 24 h in the normal population (5). Sleep includes 5 stages, stages 1–4 [non-rapid eye movement (NREM), also called slow-wave sleep (SWS) phase] and stage 5 [rapid eye movement (REM) phase]. The sleep cycle is composed of NREM, REM, and wake phases, and there are 3–5 sleep cycles each day and night. The loss of consciousness induced by anesthetics is similar to NREM sleep according to the cerebral blood flow imaging studies (6, 7). General anesthesia is a drug-induced state of loss of consciousness, which is a non-physiological process that is not easily affected by the surrounding environment. It is mainly controlled by the amount and time of anesthetic administration (8). Jiang-Xie et al. (9) found the hypothalamic neuron core group in the supraoptic nucleus and its vicinity was mainly composed of neuroendocrine cells, which were persistently and commonly activated by multiple classes of general anesthesia drug. The hypothalamus involved in sleep regulation may be the target of general anesthetics (10). The ventrolateral preoptic nucleus (VLPO) of the hypothalamus is a key area for sleep promotion that is mainly composed of GABAergic neurons. Anesthetic drugs, including propofol, and thiopental sodium, enhance GABA neuronal activity in the VLPO. These findings identified a common neural substrate underlying diverse general anesthesia drugs and natural sleep. General anesthetics induce sedation, hypnosis, and loss of consciousness by activating sleep-promoting nerve nuclei and inhibiting wake-promoting nerve nuclei in the brain (11). General anesthesia disrupts sleep/wake cycle and other circadian rhythms such as those of body temperature and melatonin secretion (12). There is some experimental evidence indicating that general anesthesia alters the molecular clock that relies on clock genes such as Period (Per), Cryptochromes (Cry), Clock and Bma1, in particular in the master clock in the suprachiasmatic nuclei (13, 14). Such a master clock controls the timing of sleep-wake cycle and circadian rhythm of body temperature and melatonin secretion, among others. Thus, a parsimonious hypothesis to explain altered sleep-wake cycle after general anesthesia involves an impaired function of the master clock (15, 16). Furthermore, Cronin et al. demonstrated that general anesthesia by inhalation (unspecified anesthetic) for gynecology surgical procedures decreased nocturnal melatonin levels on the first postoperative night compared to the second and third postoperative nights and returned to baseline levels only after the third night (17, 18). A study by Guo et al. on adults anesthetized for cardiac surgery (propofol-fentanyl) reported decreased melatonin secretion on the night after anesthesia administration (19).

## The Possible Mechanisms of Anesthetics and Analgesics

### The Effect of Anesthetics and Analgesics on Postoperative Sleep

Anesthetic-induced loss of consciousness (also termed hypnosis) could arise from anesthetic actions on the neuronal circuits regulating arousal (20). Hemmings Hugh et al. (21) reported that anesthetic drugs act at several neural sites to interrupt the connection between the cortex and its linking subcortical tissues. Opioids might cause postoperative sleep disturbances on healthy individuals and opioid addicts, which are characterized by reduced SWS, dose-dependent REM suppression, and awakenings or arousals during sleep through exerting control over various biological systems (22, 23). Furthermore, the anesthetic agent choice may affect changes in sleep architecture. Exposure of infants to propofol and remifentanil anesthesia has been shown to generally impair postoperative sleep quality (24). Studies on mice and adult humans have shown that exposure to volatile general anesthetics, including sevoflurane, isoflurane, and halothane, might cause short-term sleep disturbances and fragmentation. In humans, isoflurane anesthesia alone without surgery was shown to have no effect on REM or NREM sleep except for a shift from deeper (III and IV) to lighter (I and II) NREM sleep stages (25, 26). Pick et al. (27) found that sevoflurane inhalation can induce REM sleep deficits, delayed REM sleep recovery, and decreased latency to REM sleep, without affecting wakefulness or non-REM sleep.

### The Effect of Anesthetics and Analgesics on Immune Response and Cardiovascular Events

Opioids such as morphine may inhibit the activity of NK cell and the differentiation of T cell. It may also promote lymphocyte apoptosis and reduce the expression of toll-like receptor 4 (TLR4) on macrophages (28, 29). Fentanyl and sufentanil may decrease NK cell activity, however increase regulatory T cells (30). The side-effects of propofol are hemodynamic instability and cardiovascular complications such as hypotension, which may due to the reduced preload and afterload of the heart. Riznyk et al. found that the hypotension caused by propofol is not synchronized with heart's compensatory responses and would be intensified by high doses and high-speed injection of the drug (31, 32). The accumulated data showed that propofol inhibited the functions of neutrophils, monocytes, and macrophages in innate immunity, but not NK cells and lymphocytes. Propofol has anti-inflammatory and antioxidant effects by inhibiting innate immunity (33). Sevoflurane is one of the widely used volatile anesthetics, which may affect the immune response by increasing the levels of pro-tumorigenic cytokines and MMPs (34, 35). Similarly, isoflurane may induce apoptosis of T and B lymphocytes, attenuate NK cell activity, and decrease the Th1/Th2 ratio (36).

## Functional Changes After Postoperative Sleep Disturbances

**Figure 1:**
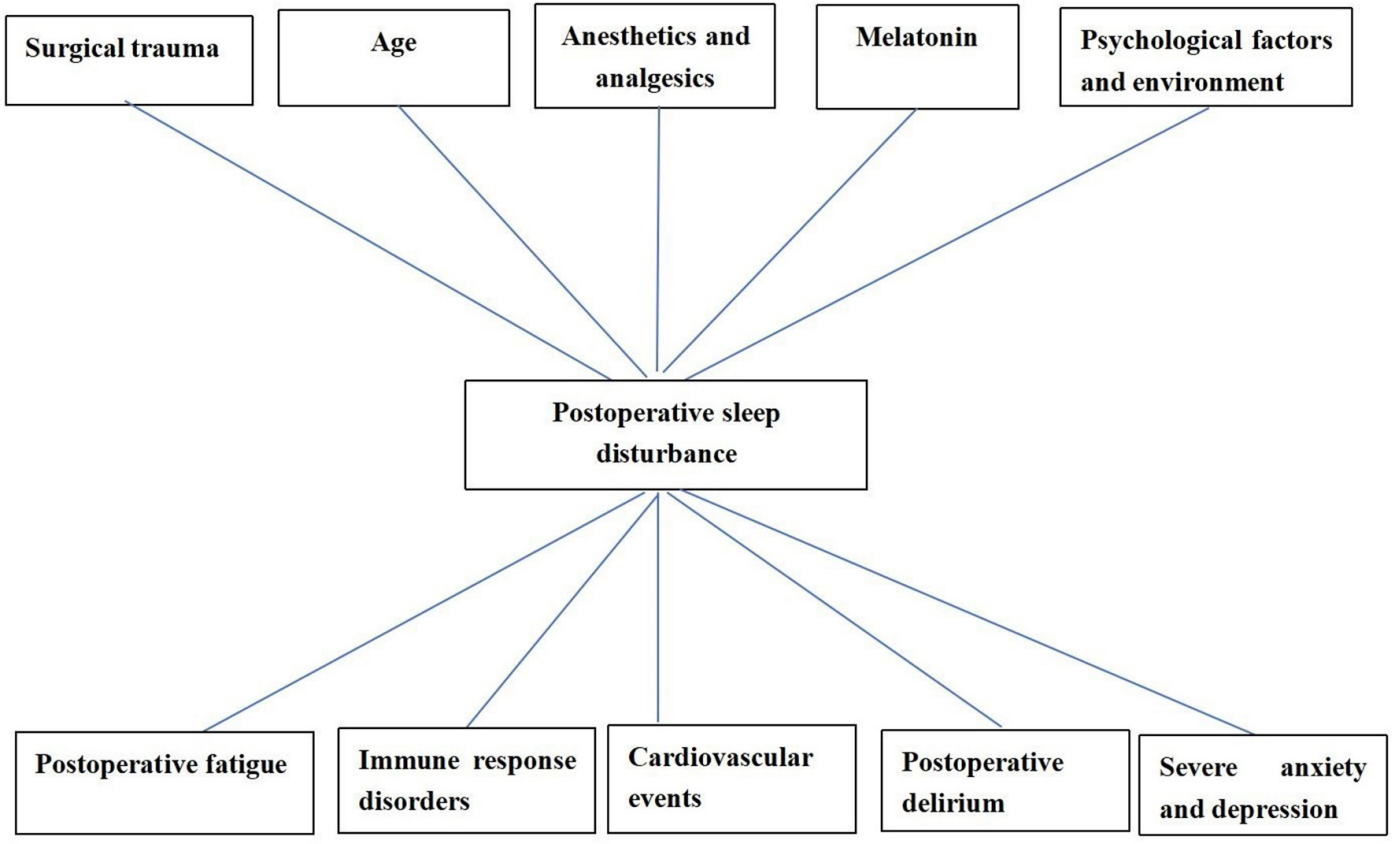
The common acupoints in the treatment of sleep disorders.

Postoperative sleep disturbances are featured by insomnia, hypersomnia and narcolepsy, changed sleep structure and increased frequency waking (37, 38). Postoperative sleep disturbances caused by general anesthesia might increase the incidence of postoperative complications such as postoperative fatigue, severe anxiety and depression, delirium (39, 40), and even severe stroke [(41); Figure 1].

### Effect of Postoperative Sleep Disturbances on Melatonin

Melatonin (N-acetyl-5-methoxytryptamine) is a methoxyindole synthesized and secreted principally by the pineal gland at night under normal light/dark conditions, which is an important physiological sleep regulator in diurnal species including humans with effects on sedation, hypnotization, and regulation of the sleep-wake cycle (42). There is a sharp increase in sleep propensity at night usually occurring 2 h after the onset of endogenous melatonin production in humans (43). Studies confirmed that large surgical traumatic stimulus and general anesthetics usually caused postoperative sleep disturbances and a higher probability of postoperative cognitive impairment (44–46). Fadayomi et al. (4) found that sleep disturbances were modifiable risk factors for postoperative delirium. Patients who had sleep disturbances before surgery were more likely to have postoperative delirium. If severe, it may result in probable mental changes [e.g., inattention, hallucination, seclusiveness, irritability, and even aggressiveness; (47)]. The degree of cognitive impairment caused by sleep disorder may range from subtle derangements in attention, reason, clarity of thought, and capacity for decision making, to confusion and delirium (48). Mehrnoush et al. (49) found that sleep disturbance was common among patients undergoing coronary artery bypass graft surgery (CABG), especially during the first week of the postoperative period, which was partly due to disturbed melatonin secretion in the perioperative period. It seemed that modifying the melatonin level by adding exogenous melatonin to the natural circadian hormone could result in the amelioration of sleep disturbance.

### Effect of Postoperative Sleep Disturbances on Immune Response and Cardiovascular Events

Sleep is thought to be an important regulator of the immune response. Sleep can regulate the function of the immune system by inducing changes in the sympathetic nervous system and hypothalamus-pituitary-adrenal axis. The circadian rhythms of hormones such as cortisol, adrenaline, and melatonin contribute to the different activities of the immune system. Sleep and immune factors are related and coordinated by inflammatory factors (tumor necrosis factors and interleukin) (50, 51). The immune system is under a bidirectional relationship with sleep. Sleep generally improve immune functions and may reduce the parameters that are involved in the rejection process. Sleep deprivation may weaken immunity and increases organism susceptibility to infection (52). These situations should adapt to the changes in sleep patterns and other parameters during the immune response to infections to which the organism is continuously exposed (53). Sleep disturbances are related to an increased risk of cardiovascular and cerebrovascular diseases (54). Insomnia may cause the development of cardiovascular disease and may increase the incidence rate of stroke and affect the poststroke outcome (55). And the treatment of sleep disturbances may improve sleep-related symptoms and benefit the long-term outcomes (56). There is also a direct correlation between the hepatic clock and Bmal1 genes and the occurrence and development of cardiovascular and cerebrovascular diseases (57). These findings have shown that circadian rhythm disturbances, which are due to the interference of general anesthetics in the biological clock genes, result in the occurrence of cardiovascular and cerebrovascular diseases via several mechanisms, thus influencing the recovery of patients.

## Risk Factors Associated With Postoperative Sleep Disturbances

**Figure 2:**
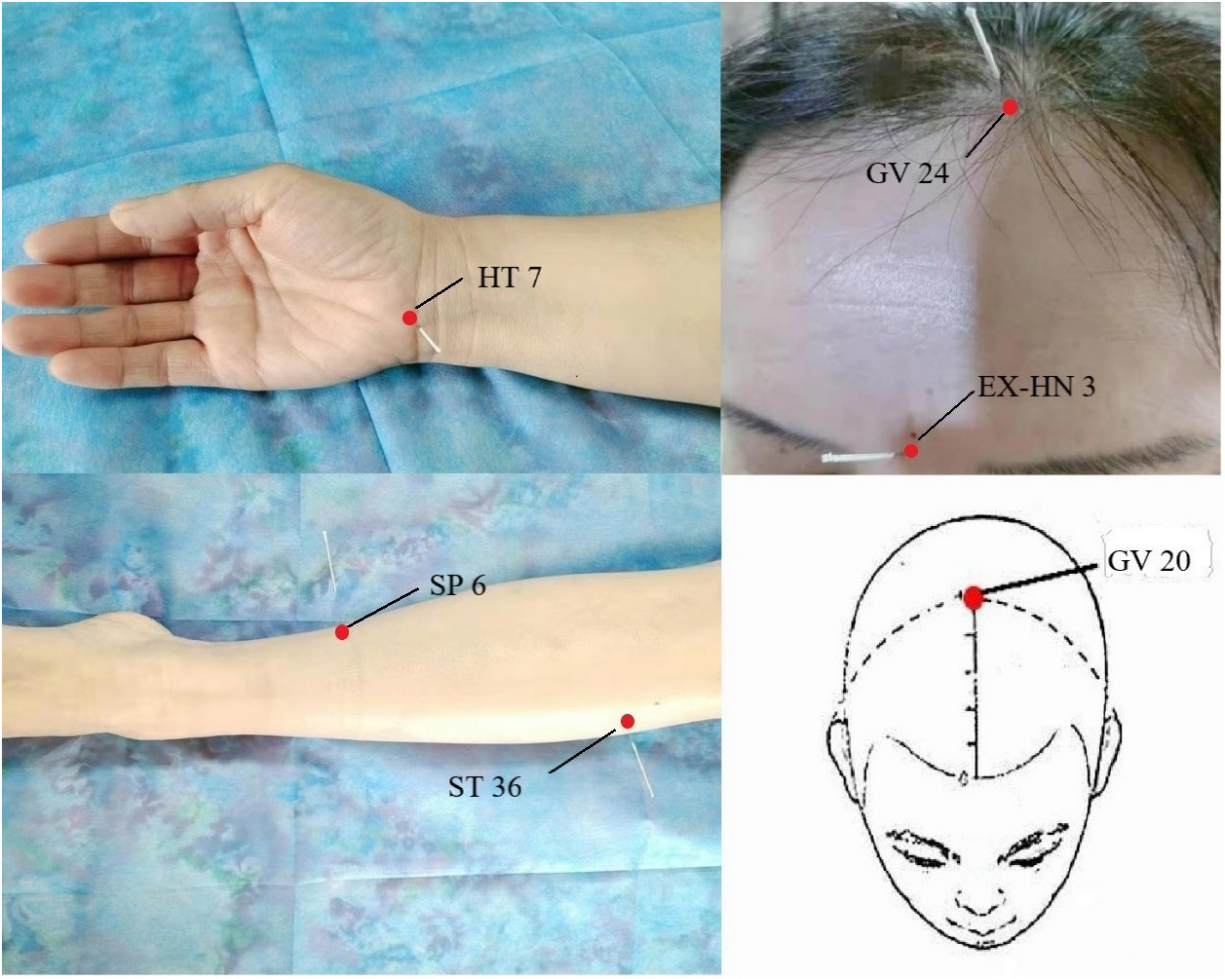
Factors associated with postoperative sleep disturbance and the adverse effects caused by postoperative sleep disturbance.

There are a variety of complicated influential factors for postoperative sleep disturbances, such as patient age, surgical trauma, anesthetic mode, postoperative pain, postoperative complications, anesthetics and analgesics, environment, and psychological factors [(39, 58); Figure 2]. People with preoperative sleep disturbances are more likely to have postoperative sleep disturbances (59). People with some diseases [such as obstructive sleep apnea (OSA), Attention-Deficit/Hyperactivity Disorder (ADHD), polycystic ovary syndrome (PCOS) often have sleep disturbances, which need our attention (60–62)]. Some studies have shown that sleep disturbances are common maladies associated with human age. The elderly are more likely to have sleep disturbances (63). The reason is that the elderly have significantly lower levels of melatonin (urine 6-methoxymelatonin) than younger people (64, 65). Further, elderly patients are a group of diminishing physiological reserves and increasing prevalence of frailty, which may affect the brain and central nervous system, leading to postoperative sleep disturbances (66). Studies indicated that the greatest incidence of sleep disturbances was found after major surgical procedures. The duration of the operative procedure was related to the duration of postoperative sleep disturbance, probably as a result of more extensive surgical trauma and a serious condition for the patients (67, 68).

This is associated with the inflammatory reaction, sympathetic nervous excitement, and endocrine disturbance caused by surgical trauma, the increase of cytokines (IL-1, IL-6, and TNF-α), cortisol and catecholamine (67, 69), and the decrease of melatonin level in the body after surgery (17). Different surgical procedures cause different complications, which have different effects on sleep quality (70). Laparoscopic surgery is associated with a reduced inflammatory response, and classic endocrine metabolic responses are either less influenced or not affected at all as compared with similar open surgeries (71). After laparoscopic cholecystectomy, only a slight change in SWS was seen on the 1st night after surgery and there was no change in REM sleep. These findings confirmed that the magnitude of the surgical trauma and/or postoperative opioid administration might be key pathogenic factors in postoperative sleep disturbances (67). Studies have shown that in general surgical and orthopedic wards, postoperative pain is the most significant and detrimental factor of postoperative sleep disturbances, which may interact with each other (72). Pain can prolong the sleep latency and reduce the total sleep time, while sleep disturbances can increase pain sensitivity and decrease the pain threshold; even the pain severity in the next day can be predicted with the postoperative sleep quality (73). The impact of psychological factors on postoperative sleep disturbances is also substantial. Postoperative patients usually feel anxious and fearful and may enter a prolonged depressed and worried state owing to the financial burden and image change, which seriously influences their sleep and rehabilitation (74). Additional factors affecting the sleep of patients are postoperative environmental change, insufficient beds, noise, and lights in the ward, electrocardiographic monitoring of vital signs, night-time treatment and nursing checks, alarms caused by machine faults, postoperative diets, and stimulation from the urinary catheter (72).

## Measures to Prevent and Improve Postoperative Sleep Disturbances

In the following section, we discussed treatment differentiating between non-pharmacological and pharmacological approaches (Table 1).

**Table 1:** Treatment of postoperative sleep disturbance.

**Non-pharmacological**	**Pharmacological**
**Environment improvement**	**Sedatives**
Reducing nursing activities	Benzodiazepines
Earplugs and eye-mask	Zolpidem
**Surgical skills improvement**	Melatonin
Reducing tissue injury	**Analgesics**
**Psychological counseling**	Opioids
**Acupuncture**	NSAIDs
Single acupuncture: sishencong (EX-HN1)	
Multiple acupuncture: baihui (GV20) yintang (GV29)	
baihui (GV20) shenting (GV24)	
neiguan (PC6) sanyinjiao (SP6) shenting (GV24)	

### Non-drug Therapy

The trauma and duration of surgeries both affected the sleep rhythm, so the severity of postoperative sleep disturbances could be effectively decreased by improving the surgical technologies and reducing tissue injury (75). The individualized anesthetic plan is recommended and local anesthesia is preferable to general anesthesia in reducing postoperative sleep disturbance (76). For patients with sleep disturbances caused by psychological factors, it is necessary to carry out health education before surgery to reduce the influence of psychological factors on sleep after surgery. Studies have shown that psychological counseling can reduce the occurrence of postoperative sleep disturbances (74). The effective measures (reducing nursing activities, using earplugs and eye masks, etc.) may minimize the impact of environmental factors on sleep (77). The efficacy of electroacupuncture alone or combined with drugs in the treatment of sleep disturbances is better than that of drugs alone and more secure (78). The common acupoints in the treatment of sleep disturbances were presented in Figure 2. Garland et al. (79) made a randomized controlled trial involving 58 breast cancer survivors experiencing bothersome hot flashes at least two times per day. Participants were randomly assigned to receive 8 weeks of electroacupuncture or daily gabapentin (total dose of 900 mg/d). By the end of treatment at week 8, the mean reduction in the Pittsburgh Sleep Quality Index (PSQI) total score was significantly greater in the electroacupuncture group than the gabapentin group. The electroacupuncture group had improved sleep duration and better sleep quality compared with baseline. The study found that electroacupuncture stimulation at bilateral Zusanli (ST36) and san yin jiao (PC6) significantly deepened the sedation level of general anesthesia in patients with propofol target controlled infusion (TCI), and provided significant delayed sedation effects (80). The combination of electroacupuncture [Shenting (GV24) and yintang (EX-HN3)] and dexmedetomidine injection are more effective than those of dexmedetomidine injection alone. Electroacupuncture appears to reduce the dose of sedative drugs required in critically ill patients, without any obvious adverse severe action, and improve the safety of treatment (81, 82). Different acupoints improve sleep through different mechanisms in electroacupuncture treatment. For example, acupuncture at baihui (GV20) for insomnia and related symptoms may be sedative by reducing the excitability of the cerebral cortex (83). Electroacupuncture at (shenmen) HT7 and (sanyinjiao) SP6 have a positive effect in improving insomnia and insomnia-induced fatigue in insomnia rats, which may be associated with its effects in up-regulating the levels of neurotransmitters [e.g., 5-HT and 5-HIAA; (84)]. Chiu et al. (85) found that acupuncture caused an increase in E2 level and a decrease in follicle-stimulating hormone (FSH) and luteinizing hormone (LH) levels, and the change of these levels was positively correlated with the improvement of sleep disturbances; meanwhile, acupuncture relieved sleep disturbances by improving the frequency and severity of hot flash. Electroacupuncture has significant analgesic and sedative effects, relieving anxiety, and reducing the risk factors of postoperative sleep disturbances (86, 87).

### Drug Therapy

For the patients who need to use drugs to relieve the postoperative sleep disturbances, zolpidem is a high-affinity positive modulator of ω1 GABAA receptors which can be used to improve sleep quality and fatigue one night before and one night after surgery (88, 89).

Melatonin as a methoxyindole synthesized and secreted principally by the pineal gland at night under normal light/dark conditions is also an effective drug for the treatment of circadian sleep-wakefulness disorder (5). The application of melatonin before and after surgery can improve sleep quality, and it can also produce a sedative effect in the early postoperative period without obvious side effects (90, 91). In addition, melatonin may be considered an effective alternative for benzodiazepines in the management of postoperative sleep disturbances (92). There is also some evidence in the literature that supports the use of melatonin in the treatment of postoperative delirium. The administration of exogenous melatonin may prevent delirium from improving postoperative sleep deprivation. The mechanism is explained under the “tryptophan theory” that the administration of exogenous melatonin might prevent delirium by inhibiting the breakthrough of both serotonin and tryptophan by the feedback mechanism (93). Clinical trials found that using dexmedetomidine intraoperatively could improve sedative effects, reduce the incidence of postoperative delirium and promote postoperative sleep quality (94, 95). Weber et al. showed that α2 adrenergic receptor agonist medications might closely produce neural activity similar to non-REM sleep (96). Dexmedetomidine may reduce the duration of mechanical ventilation, ICU stay and hospital stay among patients in the intensive care unit (ICU) (97) and may also reduce the postoperative 30-day mortality, postoperative delirium, atrial fibrillation, and cardiac arrest in patients underwent cardiac surgery (98). Though sedatives such as benzodiazepines and “Z drugs” (zaleplon, zolpidem, and zopiclone) may improve sleep, however they may also contribute to confusion and delirium especially in the elderly (99). The relationship between sleep and pain is reciprocal, poor sleep also raises the sensitivity of pain. Previous study demonstrated that opioids could improve postoperative sleep quality, duration, or efficiency. Opioids could also cause sleep disturbances which lead to hyperalgesia (100). Opioids have been widely used for analgesic effect in the operation. It also pose great challenges to the clinicians due to unfavorable side effect profiles, abuse and tolerance (101). Ketorolac tromethamine (Toradol), a non-steroidal anti-inflammatory drug, is used frequently due to its success in relieving postoperative pain and reducing the need for narcotics (102). Recent study showed that the non-steroidal anti-inflammatory drugs (NSAIDs) are not associated with increased postoperative bleeding and have fewer complications than opioids (103). When patients receive nocturnal sedative/hypnotic therapy for a long time, the sedative treatment should be reconstituted postoperatively as soon as possible to avoid postoperative sleep disturbance caused by the withdrawal of drugs (104).

## Conclusion

Postoperative sleep disturbances have severe impacts on cognition, pain perception, altered circadian rhythm, psychomotor function, cardiovascular outcomes, metabolic function, catabolic responses, and inflammation. The relationship between general anesthesia and postoperative sleep disturbances is still unclear. It is advantageous to identify patients with preexisting sleep disturbances as they are associated with a high risk of postoperative sleep disturbances. Though pharmacological treatments are helpful, they may also present some adverse effects. Non-pharmacological treatments such as electroacupuncture technology need more high-quality clinical trials with longer following-up to further investigated the efficacy.

## Author Contributions

All authors contributed to drafting or revising the article, gave final approval of the version to be published, and agree to be accountable for all aspects of the work.

## Conflict of Interest

The authors declare that the research was conducted in the absence of any commercial or financial relationships that could be construed as a potential conflict of interest.
